# Polyphenol-rich extract induces apoptosis with immunogenic markers in melanoma cells through the ER stress-associated kinase PERK

**DOI:** 10.1038/s41420-019-0214-2

**Published:** 2019-09-09

**Authors:** Karol Prieto, Yu Cao, Eslam Mohamed, Jimena Trillo-Tinoco, Rosa A. Sierra, Claudia Urueña, Tito Alejandro Sandoval, Susana Fiorentino, Paulo C. Rodriguez, Alfonso Barreto

**Affiliations:** 1 Grupo de Inmunobiología y Biología Celular, Departamento de Microbiología, Pontificia Universidad Javeriana, Bogotá, Colombia; 2 Department of Immunology, H. Lee Moffitt Cancer Center & Research Institute, Tampa, FL USA

**Keywords:** Cancer, Cell death

## Abstract

Polyphenols elicit antitumor activities, in part, through the induction of anti- or pro-oxidant effects in cancer cells which promote priming of protective anti-tumor immunity. We recently characterized a polyphenol-rich extract from *Caesalpinia spinosa* (P2Et) that stimulates in vivo antitumor responses against breast and melanoma tumor models via the promotion of immunogenic cancer cell death (ICD). However, the primary mediators whereby P2Et promotes ICD remained unknown. Here, we sought to elucidate the role that severe endoplasmic reticulum (ER) stress plays in mediating P2Et-induced apoptosis and ICD in murine melanoma cells. Our findings demonstrate a substantial selective induction of specific ER-stress mediators in B16-F10 melanoma cells treated with P2Et. While knockout of the ER stress-associated PKR-like ER kinase (PERK) prevented induction of apoptosis and expression of ICD markers in P2Et-treated cells, deletion of X-box binding protein 1 (Xbp1) did not. P2Et-driven activation of PERK in melanoma cells was found to promote ER-calcium release, disrupt mitochondrial membrane potential, and trigger upregulation of ICD drivers, surface calreticulin expression, and extracellular release of ATP and HMGB1. Notably, calcium release inhibition, but not targeting of PERK-driven integrated stress responses, prevented P2Et-induced apoptosis. Collectively, these results underline the central role of PERK-directed calcium release in mediating the antitumor and immunogenic actions of P2Et in melanoma cells.

## Introduction

Effective natural products-based therapies represent a promising strategy for the treatment of cancer and as adjuvants for immunotherapy^1^. P2Et, a polyphenol-rich extract obtained from plant *Caesalpinia spinosa*, promotes antitumor activities through the induction of immunogenic cancer cell death (ICD)^2,3^. ICD is a process characterized by the generation of DAMPs (surface expression of calreticulin (Ecto-CRT); ATP and HMGB1 release) and subsequent DAMPs mediated immune system activation^4,5^. Consequently, vaccination with P2Et-treated cancer cells can induce anti-tumor responses in breast and melanoma tumor-bearing mice via ICD-mediated induction of protective immunity^2,3^.

Appropriate cellular stress responses are necessary for the production of danger signals during ICD, including the activation of mediators related to endoplasmic reticulum (ER) stress and autophagy^5,6^. Several mechanisms triggered by natural products-based therapies have been shown to alter cellular homeostasis through alterations in calcium (Ca^2+^) levels, oxidative stress, hypoxia, or glucose/nutrient deprivation. These alterations in homeostasis disturb the protein-folding capacity of the ER, and consequently induce activation of the unfolded protein responses (UPR)^7^. UPR induction is characterized by the activation of three ER membrane sensors: the activating transcription factor 6 (ATF6), the inositol-requiring enzyme 1 (IRE1α), and the PKR-like ER kinase (PERK)^5,8^ with the ultimate goal of restoring ER homeostasis. Together, these mediators restore the folding capacity of the ER, thereby promoting adaptative processes enabling cell survival. Despite this pro-survival function, dysregulated activation of the UPR in cancer cells can lead to several forms of cell death^5,8–10^. During UPR, PERK kinase is activated by dimerization and autophosphorylation; PERK subsequently phosphorylates the eukaryotic translation initiation factor 2α (eIF2α) to thereby induce integrated stress responses (ISR) that inhibit the translation of most mRNAs, while promoting the expression of ATF4 and the pro-apoptotic transcription factor C/EBP-homologous protein (CHOP)^7^. Additionally, PERK activation regulates mitochondria–ER contact flux sites (mitochondria-associated membranes (MAMs)), which play a primary role in cellular adaptation to elevated levels of reactive oxygen species (ROS) and Ca^2+^^11,12^. Thus, PERK-associated signaling can promote either pro- or anti-survival outcomes in a context dependent manner.

Polyphenols are a large group of natural products that can exhibit antitumor activities by modulating Ca^2+^ levels, ROS production, ER stress, and autophagy^13,14^. Modulation of Ca^2+^ and ROS levels can provoke ER-stress responses, enable generation of DAMPs and anti-tumor immunity by ICD inducers such as anthracyclines and hypericin-photodynamic therapy (Hyp-PTD)^15^. Indeed, ER stress and PERK are necessaries for Ecto-CRT during ICD, hence EIF2α phosphorylation has emerged as a pathognomonic marker in ICD^16,17^. Conversely, ROS has been implicated in induction of tolerogenic responses in the tumor microenvironment^15,18–20^. We recently described that P2Et, a polyphenol-rich extract, induces ICD in B16-F10 cells^2^. However, it remains unknown the mechanisms by which polyphenols like those in P2Et evoke DAMPs during ICD. In this study, we sought to determine in vitro the impact of ER stress induction and ICD activation induced by P2Et treatment on melanoma B16-F10 cells. Results show that PERK-driven Ca^2+^ release as opposed to PERK-induced ISR plays a primary role in mediating P2Et-induced apoptosis and in the generation of ICD-associated DAMPs in a ROS independent manner. Collectively, our findings suggest that P2Et induces ICD in a distinct manner that promotes its potential therapeutic use as a natural product to eliminate cancer cells, boost protective anti-tumor immune responses, and enhance the effectiveness of immunotherapy.

## Results

### Polyphenol-rich extract induces apoptosis and ER stress in melanoma cells

**Fig. 1:**
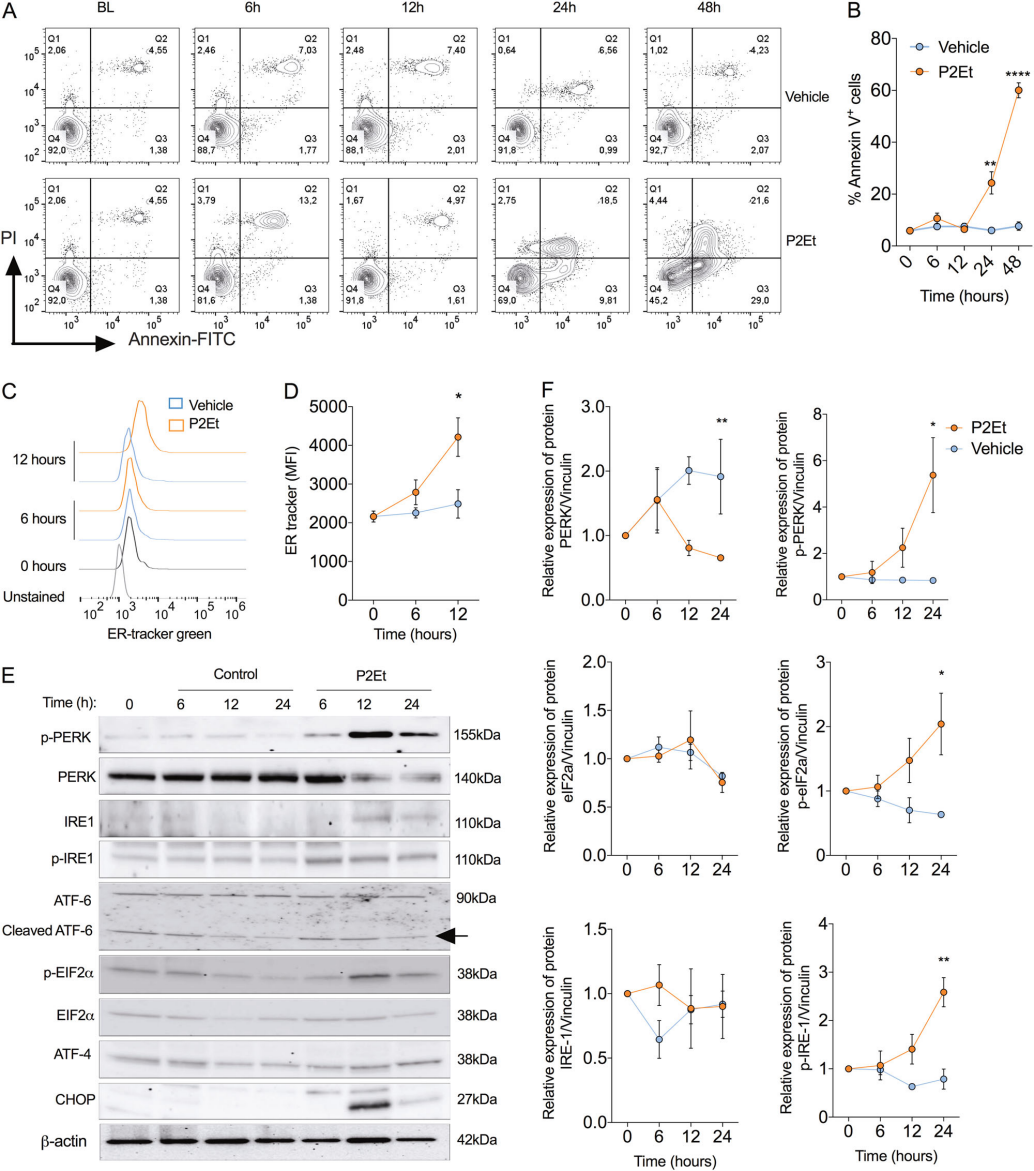
P2Et extract-induced apoptosis and endoplasmic reticulum stress in a time dependent manner on B16-F10 cells. **a** Apoptosis was analyzed by flow cytometry using Annexin V-FITC and PI stain. Representative contour plot of B16-F10 cells incubated with P2Et IC50 (74.7 µg/ml) for 6, 12, 24, or 48 h or Vehicle (ETOH) is shown. Baseline (BL) correspond to B16-F10 cells at 0 h. **b** The percentages of Annexin V positive cells (early and late apoptotic cells) were expressed as mean ± SEM for three independent experiments. **c** Cells were collected and labeled with ER-Tracker green 100 nM. Representative histogram of B16-F10 cells treated with vehicle or P2Et IC50 at 6 or 12 h is shown. **d** The geometric means of the fluorescence intensity (MFI) were expressed as mean ± SEM from three independent experiments. **e** Representative image of UPR proteins expression by western blot in B16-F10 cells treated with vehicle or P2Et IC50 for 6, 12, or 24 h. β-actin protein was used as a loading control. **f** Quantitative assessment of band intensity was performed by Image Lab 6.0 software using data from three independent experiments. ^*^P < 0.05; ^**^P < 0.01; ^***^P < 0.001

We aimed to elucidate the cytotoxic effects of P2Et on the melanoma cell line B16-F10. The induction of the apoptosis was evidenced by phosphatidylserine (PS) externalization using the marker annexin V. Time-dependent increase of annexin V^+^ cells was observed in B16-F10 cells treated with P2Et compared to cells treated with vehicle control (Fig. 1a, b). In addition, significant induction of ER stress, as indicated by an elevated ER-tracker fluorescence (Fig. 1c, d) and heightened phosphorylation of the UPR drivers PERK and IRE-1α (Fig. 1e, f), occurred in B16-F10 cells exposed to P2Et compared to controls. In agreement with significant activation of PERK signaling, P2Et treatment enhanced expression of eIF2α phosphorylated (p-eIF2α) and CHOP (Fig. 1e, f). These results suggest the co-induction of apoptosis and ER stress in melanoma cells upon treatment with P2Et.

**Fig. 2:**
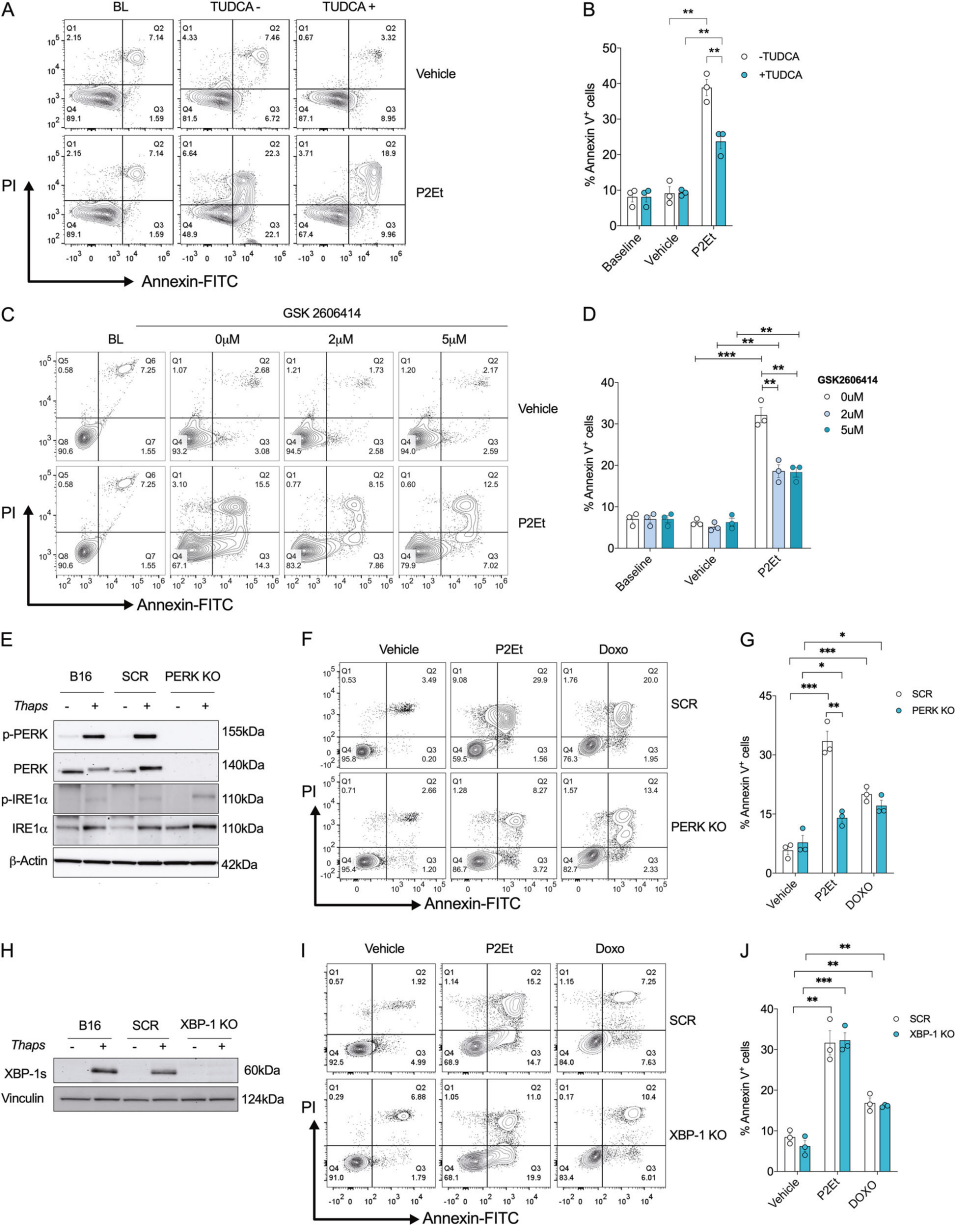
Inhibition of endoplasmic reticulum stress and PERK on B16-F10 cells decreases apoptosis induction by P2Et. B16-F10 cells were treated with P2Et IC50 or Vehicle for 24 h after pretreatment with TUDCA or PERK kinase activity inhibitor (GSK2606414) for 2 h. **a** A representative contour plot of B16-F10 cells pretreated with 0.5 mM TUDCA and labeled with Annexin V-FITC and PI is shown. **b** Percentages of Annexin V positive cells were expressed as mean ± SEM of three independent experiments. **c** A representative contour plot of B16-F10 cells pretreated with PERK inhibitor GSK2606414 (2–5 µM) and labeled with Annexin V-FITC and PI is shown. **d** The percentages of apoptotic cells are shown in bars of media ± SEM (three independent experiments). **e** Representative western blot analysis of PERK and IRE-1 total and phosphorylated proteins in SCR and PERK KO clones treated or not with thapsigargin is shown. β-actin was used as a loading control. **f** A representative contour plot of SCR and PERK KO clones treated with P2Et IC50 (74.7 µg/ml) or Vehicle for 24 h and labeled with Annexin V-FITC and PI is shown. **g** Percentages of Annexin V positive cells were expressed as mean ± SEM of three independent experiments. **h** Representative western blot analysis of spliced XBP-1 in SCR and XBP-1 *knockout* (XBP-1 KO) clones is shown in B16-F10 cells treated or not with thapsigargin. Vinculin was used as a loading control. **i** A representative contour plot of SCR and XBP-1 *knockout* (XBP-1 KO) clones treated with P2Et IC50 (74.7 µg/ml), Doxorubicin (DOXO, 0.06 µg/ml) or Vehicle for 24 h and labeled with Annexin V-FITC and PI is shown. **j** Percentages of Annexin V positive cells were expressed as mean ± SEM of three independent experiments. ^*^P < 0.05; ^**^P < 0.01; ^***^P < 0.001

To determine the role of ER stress in mediating induction of apoptosis by P2Et, we pretreated cells with TUDCA, a chemical chaperone that blocks the activation of ER-stress mediators^21^, prior to P2Et application. Cells pre-treated for 2 h with TUDCA prior to 24 h P2Et exposure exhibited attenuated PS externalization, indicating that P2Et-induced apoptosis is partially mediated by ER stress (Fig. 2a, b). Accordingly, we observed a decrease in caspase 3 and 7 cleavage after pre-treatment with TUDCA and P2Et (Fig. S1A). Next, to specifically assess the role of PERK we use GSK2606414, a small molecule inhibitor of PERK-phosphorylation^22^. Application of GSK2606414 prior to P2Et treatment corresponded to a significant decrease in PERK-phosphorylated (p-PERK) and a lower percentage of annexin V^+^ cells (Figs. S1B, 2c, d). To confirm the role of PERK in P2Et-mediated apoptosis, we generated *Perk*-deficient B16-F10 cells by using *Perk* specific (PERK KO) or scramble control (SCR) CRISPR/Cas9 constructs (Fig. 2e). Notably, elimination of PERK did not alter the activation of IRE-1α after treatment with thapsigargin (Fig. 2e), suggesting our PERK knockout system enabled selective inhibition of only the PERK branch of the UPR. Remarkably, PERK deletion blocked the induction of apoptosis in B16-F10 cells treated with P2Et as compared to controls. However, similar apoptosis levels were detected in PERK-deficient and SCR B16-F10 cells after treatment with PERK-independent apoptosis inducer doxorubicin (DOXO) (Fig. 2f, g). Next, we used CRISPR/Cas9 generated *Xbp-1 knockout (*XBP-1 *KO)* B16F10 cells to determine whether silencing of the IRE-1-XBP1 branch of the UPR impacted the induction of apoptosis by P2Et. A similar induction of apoptosis was observed in B16-XBP*-*1 KO and control cells treated with P2Et or DOXO for 24 h (Fig. 2h–j). Taken together, our results indicate that apoptosis induced by P2Et is dependent on ER-stress induction and PERK activation.

### Apoptosis induced by P2Et is independent of the integrated stress response and ROS production

**Fig. 3:**
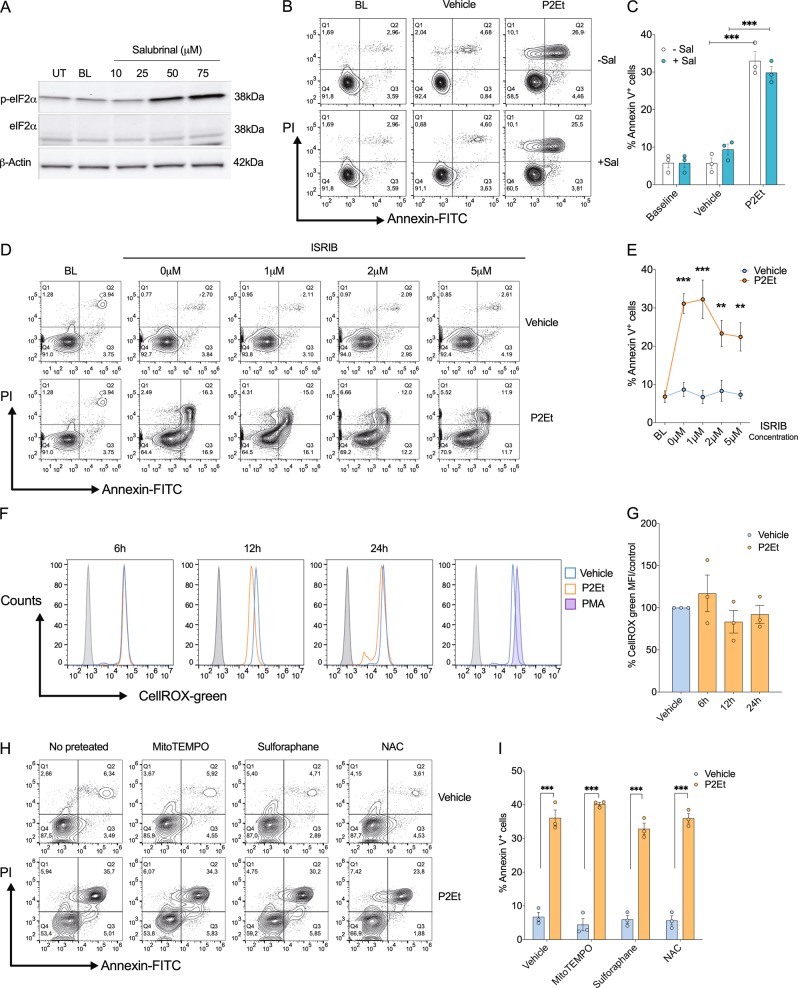
Inhibition of integrative stress response and ROS production does not affect apoptosis induction by P2Et on B16-F10 cells. B16-F10 cells were 2 h pre-treated with salubrinal or ISRIB and then treated with P2Et IC50 (74.7 µg/ml) or Vehicle for additional 24 h. **a** A representative image of eIF2a total or p-eIF2α analysis by western blot of B16-F10 cells pretreated with several concentrations of salubrinal (10, 25, 50, and 75 µM). β-actin was used as a loading control. **b** A representative contour plot of B16-F10 cells pretreated with 75 µM Salubrinal, treated with P2Et or Vehicle and labeled with Annexin V-FITC and PI is shown. **c** Percentages of Annexin V positive cells were expressed as mean ± SEM of three independent experiments. **d** A representative contour plot of B16-F10 cells pretreated with several concentrations of ISRIB (1, 2, and 5 µM) and treated with P2Et or vehicle for additional 24 h. **e** Percentages of Annexin V positive cells were expressed as mean ± SEM of three independent experiments. **f** B16-F10 cells were treated with P2Et IC50 or Vehicle for 6, 12, and 24 h, following cells were harvested and labeled with 100 mM CellROX green. A representative histogram is shown. **g** Percentage folding change of CellROX MFI from treated cells relative to the vehicle from three independent experiments is shown. **h** B16-F10 cells were pre-treated 2 h with antioxidants (2 mM mitoTEMPO, 2 mM sulforaphane, and 2.5 mM N-acetyl-cysteine-NAC), and then treated with P2Et IC50 (74.7 µg/ml) or Vehicle for additional 24 h. A representative contour plot of B16-F10 cells stained with Annexin V-FITC and PI is shown. **i** Percentages of Annexin V positive cells were expressed as mean ± SEM of three independent experiments. ^*^P < 0.05; ^**^P < 0.01; ^***^P < 0.001

Activation of PERK after ER stress drives ISR through the phosphorylation of eIF2α and the subsequent induction of ATF-4 and CHOP^23^. To test whether ISR contributes to P2Et-induced apoptosis, we used salubrinal (Sal), an inhibitor of eIF2α dephosphorylation^24^ (Fig. 3a–c), and the ISR inhibitor (ISRIB), an inhibitor of eIF2α phosphorylation^25^ (Fig. 3d, e). Apoptosis induced by P2Et was not altered upon pre-treatment with Sal or ISRIB, suggesting that P2Et directs apoptosis in B16-F10 cells in an ISR-independent manner. To validate our results, we stably transfected B16-F10 cells with eIF2α plasmids coding for either a wild type form of eIF2α (eIF2α-S51S), a dominant negative phosphorylation resistant form of eIF2α (eIF2α-S51A) in which serine 51 has been mutated to alanine, or a phosphomimetic form of eIF2α (eIF2α-S51D) in which serine is mutated to aspartate. Genetic modulation of eIF2a activity did not impact the rate of apoptosis induction in P2Et-treated B16-F10 cells (Fig. S2A, B). In agreement, inhibition of *Chop* using an antisense oligonucleotide did not affect apoptosis induced by P2Et treatment (data not shown). These findings suggest that ISR induction plays little to no role in mediating the effects of P2Et and that an alternative pathway, but not canonical PERK activation is necessary for P2Et induced apoptosis in melanoma cells.

Polyphenols and other antioxidants such as curcumin or resveratrol induce apoptosis through an accumulation of ROS or modulation of glucose uptake that can trigger activation of the ER stress response^13,26,27^. Consequently, we sought to determine whether P2Et-triggered apoptosis in B16-F10 cells could be mediated by ROS accumulation. Unexpectedly, treatment of B16-F10 cells with P2Et did not alter ROS levels in B16-F10 cells; consequently, application of three different antioxidants (MitoTEMPO, sulforaphane, and NAC) prior to P2Et exposure did not prevent P2Et-induced apoptosis (Fig. 3f–i).

### PERK signaling and Ca^2+^ increase induced by P2Et is essential to mitochondrial dysfunction and apoptosis generation

**Fig. 4:**
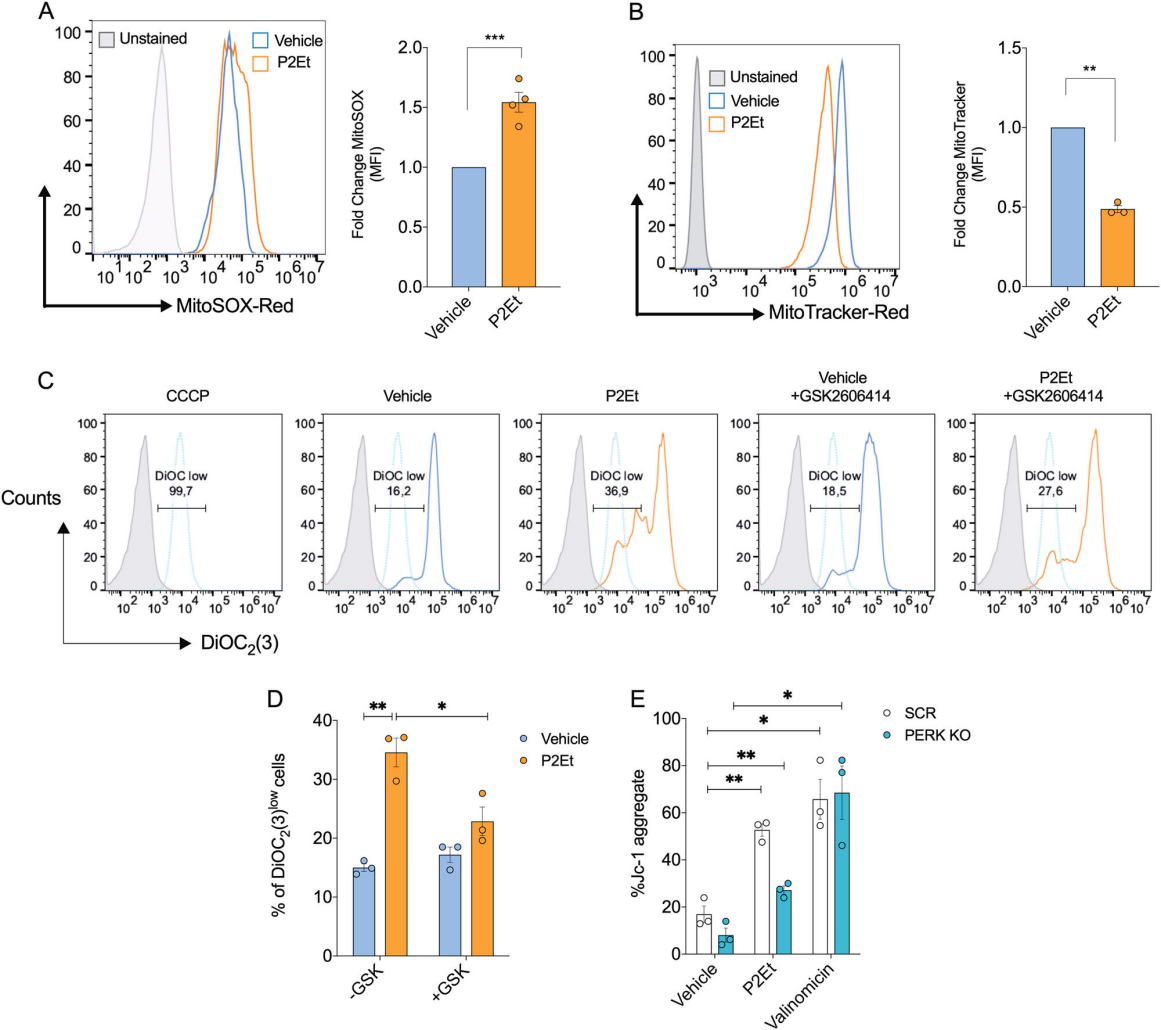
Inhibition of PERK decreases mitochondrial dysfunction induced by treatment of B16-F10 cells with P2Et. B16-F10 cells were treated with P2Et IC50 or Vehicle for 24 h, and then cells were harvested and labeled with **a** MitoSOX Red (100 mM) or **b** Mitotracker Red (100 mM). Fold change was determined using MFI from P2Et treatment relative to the vehicle for both dyes. Evaluation of mitochondrial membrane potential (ΔΨm): **c** B16-F10 cells were pre-treated with GSK2606414 (5 µM) for 2 h and then treated with P2Et IC50 or vehicle for additional 24 h. Cells were harvested and labeled with DioC2(3), the gate to analyze was made over B16-F10 cells treated with 1 µM of carbonilcianuro-m-clorofenilhidrazona (CCCP) 5 min prior FACS. A representative histogram of DioC2(3) fluorescence is shown. **d** Percentage of DioC2(3) low cells expressed as mean ± SEM of three independent experiments is shown. **e** JC-1 staining analysis of SCR and PERK KO cells treated for 12 h with P2Et IC50 or vehicle, % of Jc-1 aggregates expressed as mean ± SEM of three independent experiments is shown. ^*^P < 0.05; ^**^P < 0.01; ^***^P < 0.001

ER stress-mediated apoptosis has been associated with mitochondrial dysfunction and the subsequent cytoplasmic release of pro-apoptotic signaling molecules such as cytochrome c that ultimately lead to caspase activation. Our previous report showed that P2Et increased the release of mitochondrial cytochrome c in B16-F10 cells, which correlated with cleavage of caspase 3 and 9^2^. Consistent with this data, we found that P2Et disrupted mitochondrial homeostasis, as demonstrated by an increase in mitochondria ROS content (MitoSOX), a decrease in mitochondrial mass (MitoTracker) and reduction of mitochondrial membrane potential (Fig. 4). Elimination or inhibition of PERK via GSK2606414 or use of B16-F10 PERK KO cells prevented P2Et-induced mitochondrial dysfunction while the inducer of loss of mitochondrial membrane potential, valinomycin, altered mitochondrial membrane in both PERK KO and SCR cells (Fig. 4c–e). In addition, we observed a decrease in mitochondrial oxygen consumption rate after P2Et treatment, which was normalized upon elimination of PERK (Fig. S3A). Together, these results show that PERK signaling induced by P2Et is essential in mediating treatment induced mitochondrial dysfunction and apoptosis induction.

**Fig. 5:**
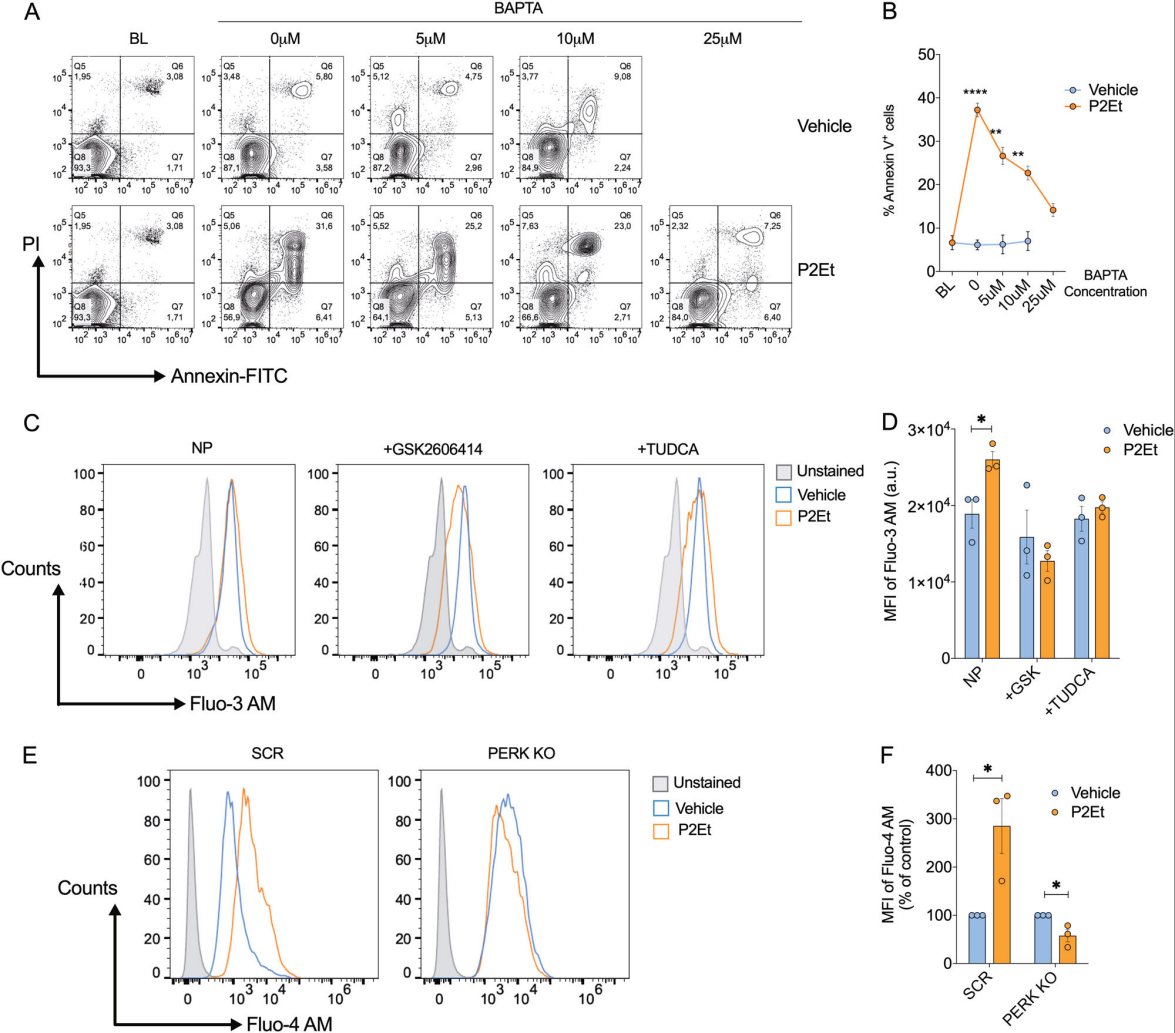
Inhibition of ER stress and PERK decrease intracellular calcium levels in P2Et treated B16-F10 cells. **a** B16-F10 cells pretreated with different concentrations of BAPTA (5, 10, and 25 µM) and treated with P2Et or vehicle for additional 24 h. A representative contour plot of stained cells with Annexin V-FITC and PI is shown. **b** Percentages of Annexin V positive cells were expressed as mean ± SEM of three independent experiments. **c** B16-F10 cells were pre-treated GSK2606414 (5 µM) or TUDCA (0.5 mM), or non-pretreated (NP) for 2 h and then treated with P2Et IC50 or vehicle for additional 24 h. Cells were harvested and labeled with calcium dye Fluo-3 AM (150 mM). A representative histogram showing Fluo-3 AM fluorescence is shown. **d** MFI of Fluo-3 AM expressed as mean ± SEM of three independent experiments is shown. **e** SCR and PERK KO cells treated for 24 h with P2Et IC50 or vehicle were stained with Fluo-4 AM (1.5 μM). A representative histogram is shown. **f** Percentage folding change of Fluo-4 AM MFI from treated cells relative to vehicle treatment expressed as mean ± SEM of three independent experiments is shown. ^*^P < 0.05; ^**^P < 0.01; ^***^P < 0.001; ^****^P < 0.0001

Recent studies show that PERK regulates the maintenance of homeostatic levels and flux of calcium between the ER and mitochondria^11^. Notably, elevation in cytosolic Ca^2+^ has been linked to induction of apoptosis and production of DAMPs during ICD^11,28^. To determine what role Ca^2+^ accumulation plays in P2Et induced apoptosis, we applied the Ca^2+^ chelator BAPTA prior to P2Et exposure. We observed a dose-dependent decrease in apoptosis when P2Et-exposed B16-F10 cells were pretreated with BAPTA (Fig. 5a, b). In agreement, BAPTA pretreatment of P2Et-exposed cells decreased the percentage of DioC2(3)^low^ cells further supporting the major role of the P2Et-mediated mitochondrial Ca^2+^ accumulation in mediating treatment induced apoptosis of B16F10 cells (Fig. S3B, C). Next, we compared cytoplasmic Ca^2+^ levels in B16-F10, SCR, or PERK KO cells using Fluo3/Fluo-4. P2Et-treated cells showed higher cytoplasmic Ca^2+^ levels; Ca^2+^ returned to basal levels by the addition of the ER stress inhibitor TUDCA or upon the inhibition or elimination of PERK (Fig. 5c–f). Collectively, our results suggest that P2Et-induced activation of PERK drives release of Ca^2+^ from the ER to direct mitochondrial dysfunction and ultimately induction of apoptosis.

### P2Et induce Ecto-CRT, and ATP and HMGB1 release in a PERK-dependent manner

**Fig. 6:**
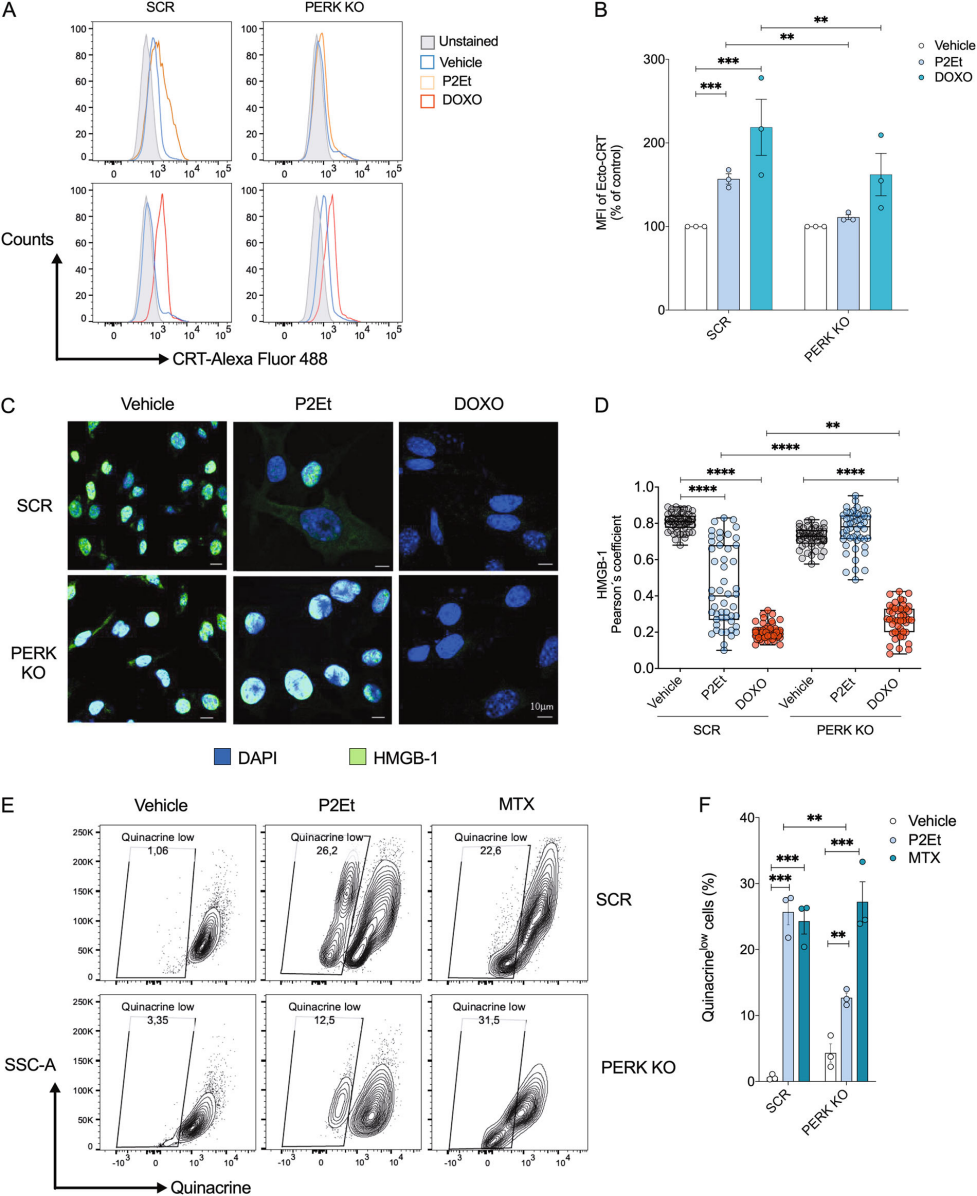
Inhibition of PERK decreases DAMPs generation in B16F10 cell treated with P2Et. **a** SCR or PERK KO clones were treated with P2Et IC50, DOXO IC50, or vehicle for 12 h. Surface exposure of CRT was determined by flow cytometry among viable cells (Aqua negative) and primary antibody for CRT (rabbit anti-mouse) was detected using conjugate goat anti-rabbit secondary antibody Alexa Fluor 488. A representative histogram is shown. **b** Percentage folding change of MFI from treated cells relative to vehicle treatment expressed as mean ± SEM of three independent experiments is shown. **c** SCR or PERK KO clones were treated with P2Et IC50, DOXO IC50 or with vehicle for 48 h. Primary antibody for HMGB1 was detected using Alexa Fluor 488 conjugated goat anti-rabbit secondary antibody (green) and DAPI (blue) for the nuclei. Images were acquired with confocal microscope Olympus FV1000 with an oil 60× PlanAPO objective. Representative images of three independent experiments are shown. White bar corresponds to 10 μm **d**. Scatter plot of Pearson coefficients was assessed in 50 cells for each treatment in 3 independent experiments. Results from one representative experiment expressed as means ± S.E.M are shown. **e** SCR or PERK KO clones were treated with P2Et IC50 or 0.5 μg/ml MTX for 48 h. After treatment cells were stained with quinacrine (1 μM) and PI. Quinacrine low cells were determined among viable cell (PI-negative cells). A representative dot plot for each treatment is shown **f** Percentage of quinacrine low cells are expressed as mean ± SEM of three independent experiments. ^*^P < 0.05; ^**^P < 0.01; ^***^P < 0.001, ^****^P < 0.0001

We previously demonstrated that B16-F10 cells treated with P2Et increase expression of DAMPs associated with ICD including Ecto-CRT, ATP, and HMGB1^2^. In addition, Ecto-CRT has been reported to occur as a consequence of PERK-mediated ER stress^6,29^. Thus, we assessed whether PERK was involved in P2Et-treatment associated Ecto-CRT. Therefore, we evaluated Ecto-CRT in SCR and PERK KO cells treated with P2Et or ICD control (DOXO). SCR cells treated with P2Et or DOXO increased Ecto-CRT, compared to vehicle-treated cells. Conversely, Ecto-CRT decreased in PERK KO cells treated with P2Et and DOXO (Fig. 6a, b) demonstrating the role of PERK in directing tumor cell expression of Ecto-CRT^6^. To investigate HMGB1 and ATP release we assessed nuclear translocation of HMGB1^30^ and the percentages of low-quinacrine cells (a fluorescent marker of intracellular ATP storage sites)^31^ in P2Et treated groups. Interestingly the absence of PERK corresponded to a decrease in both HMGB1 nuclear delocalization (increasing Pearson’s coefficient) and the percentage of low-quinacrine cells after P2Et treatment. By comparison, HMGB1 and ATP release mediated by conventional ICD inducers DOXO and mitoxantrone (MTX) were not dependent on PERK (Fig. 6c–f). Collectively, these results suggest that P2Et induces expression of ICD associated DAMPs: Ecto-CRT, and ATP and HMGB1 release in a PERK-dependent manner.

## Discussion

**Fig. 7:**
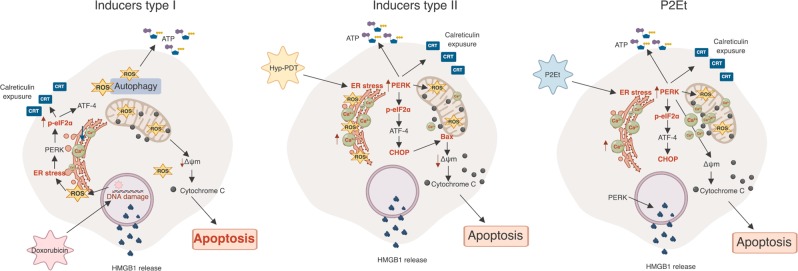
Proposed model for P2Et effect on B16-F10 tumor cells. **a** Inducers type I as doxorubicin induces DNA damage and ROS production, therefore, increase ER stress and autophagy associated with Ecto-CRT and ATP release, respectively. In addition, apoptosis is independent of PERK. **b** Inducers type II as Hyp-PDT increase ROS in ER and induce Ecto-CRT, ATP release and apoptosis in a PERK-dependent manner. In both types of ICD inducers, CRT exposure is dependent on ER-calcium levels depletion. **c** On the other hand, P2Et increase ER stress signaling via PERK promoting an increase in ER and cytoplasmic Ca^2+^ levels. This calcium will be in part responsible for the induction of mitochondrial damage and apoptosis without ROS participation. Parallel, P2Et also induced the ICD markers Ecto-CRT, and HMGB1 and ATP release in a PERK-dependent manner. Image created using BioRender (https://biorender.com/)

Therapies derived from natural compounds have demonstrated antitumor potential along with a low risk of side effects and possess more tolerable toxicity profiles as compared to conventional antitumor drugs. Plant extracts contain a diverse range of compounds including polyphenols which may exert their therapeutic functions by increasing ER stress, inducing oxidative stress or promoting apoptosis of tumor cells^14,32^. For these reasons, natural compounds have recently been considered as resource for development of new chemotherapeutic drugs or chemosensitizing agents^33,34^. Our work on P2Et, a characterized polyphenol-rich extract from *C. spinosa* plant^35^, demonstrates the therapeutic value of plant derived therapies. P2Et induces apoptosis and ICD to promote expression of immunogenic markers, including CRT, HMGB1, and ATP in 4T1 cells^3^ and B16-F10 cells^2^. Vaccination with P2Et-treated cells induced an immune mediated reduction in tumor volume; while immunocompetent mice exhibited decreased tumor growth the tumor-protective effect was abolished in immunodeficient mice^2^. Consistent with these results, we show here that in our in vitro B16-F10 melanoma model P2Et promotes apoptosis in an ER stress dependent manner. P2Et treatment induced noncanonical PERK-activation and induced apoptosis in a eIF2α and CHOP independent manner^36^. Interestingly, similar to other ICD inducers, PERK was necessary for P2Et induced elevation of Ecto-CRT and ATP release^6,37^, but interestingly PERK also was necessary for HMGB1 release indicating the need for further research into the pathways involved in P2Et-induced tumor ICD (Fig. 7).

Although UPR has been established to restore cellular homeostasis and relieve ER stress to promote cell survival, under prolonged and severe ER stress the UPR can become cytotoxic rather than cytoprotective^7,38^. Our assessment of P2Et-induced UPR signaling revealed that P2Et-induced apoptosis is dependent on PERK but not IRE-1α/XBP-1. ATF-6 and ATF-4 have been reported to be involved in the survival of melanoma cells, yet P2Et treatment was not observed to impact their expression^39,40^. Although we observed an increase in p-eIF2α and CHOP expression after P2Et treatment neither of these molecules were involved in P2Et-induction of apoptosis. While the antitumor activity of many other polyphenols has been associated with ROS accumulation and ER stress^14,32^, our results showed that ROS production was not increased in P2Et treated B16-F10 cells. The finding that antioxidants did not rescue P2Et-induced apoptosis further indicates that P2Et mediated tumor killing occurs in an ROS independent manner.

Otherwise, ER stress has been shown as a determinant mechanism for ICD induction, because contributes with apoptosis and DAMPs induction in tumor cells^5,7^. Consequently, inducers of ICD can be classified according to the contribution and quality of ER stress^5^. Inducers type I as anthracyclines, mitoxantrone, and oxaliplatin increase ROS production after DNA damage to induce apoptosis and enhance ER stress eliciting Ecto-CRT exposure depending on PERK; while additional DAMPs (ATP and HMGB1) are released by independent mechanisms^5,41^. Conversely, inducers type II as Hyp-PTD increase ROS production directly in ER compartment stimulating Ecto-CRT, ATP release and apoptosis through ER stress in a PERK-dependent manner. Like inducers type I, Hyp-PDT release HMBG-1 by a passive mechanism after cell death^37,41^. In both, type I and type II inducers, the ER-Ca^2+^ depletion is evoked in response to ROS increase and it is required for Ecto-CRT^11,28^. In contrast with other ICD inducers, apoptosis mediated by P2Et is dependent on ER stress, which increases cytoplasmic levels Ca^2+^, but independent of ROS production. Interestingly, p-PERK is required for Ecto-CRT, ATP, and HMGB1 release. Even though, HMGB1 release in ICD has not been associated with PERK, some studies showed a role of Ca^2+^ levels in this process^42^. Thus, it is plausible that P2Et induction of HMGB1 release depends of Ca^2+^ increase after ER stress generation.

P2Et could be advantageous in respect to other ICD inducers, because increasing ROS in tumor milieu may induce ER stress in infiltrating immune cells leading to a decreased immune response^43,44^. In addition, the stressed tumor cells can transfer ER-stress factors toward MDSC and dendritic cells favoring UPR activation and immunosuppression activities in these cells^44,45^. P2Et induction of UPR and apoptosis without ROS increase, suggests a role for P2Et in the reduction of tumor microenvironment immunosuppression induced by stressed tumor cells. In agreement with that, we recently showed in a transplantable model of melanoma B16-F10 cells, that P2Et induced reduction of tumor growth that corresponded with a decrease in tumor-infiltrating MDSCs^46^.

On the other hand, Ca^2+^ is an important second messenger in cells and is linked with regulation of several cell death and survival pathways, including UPR, autophagy, apoptosis, proliferation, and migration. Thus, Ca^2+^ has begun to be considered as a therapeutic target in cancer. Modulation of Ca^2+^ levels is used by natural products to induce apoptosis in cancer cells^47,48^. Some less toxic natural products like resveratrol increase cytoplasmic and mitochondrial Ca^2+^ levels by modulation of Ca^2+^ transporters/channels/pumps specifically in cancer cells^49,50^. The lethal increase of Ca^2+^ by natural compounds would activate multiples Ca^2+^ dependent pathways which together would be more efficient inducing cell death. In addition, the role of Ca^2+^ in immunogenic cell death is also relevant, studies showed that anthracyclines and Hyp-PDT modulate the decrease of Ca^2+^ in the ER lumen and this Ca^2+^ depletion is required to Ecto-CRT^28,41^. In spite of that, the role of Ca^2+^ in HMGB1 release is not clear, and a recent study with a new natural product suggested that an increase in cytoplasmic Ca^2+^ levels was involved with HMGB1 release^42^. In the other hand, MAMs are the main mechanism for regulation of Ca^2+^ pulses into mitochondria during ER stress promoting cell survival. However, during sustained ER stress, PERK plays an important role in MAMs conformation, Ca^2+^ cell signaling and apoptosis^11,47^. Here, we showed that Ca^2+^ chelator BAPTA blocked P2Et-induced apoptosis and mitochondrial dysfunction showing the role of Ca^2+^ in P2Et induction of cell death and suggesting a role in ICD. In addition, PERK is necessary for P2Et increases of cytoplasmic Ca^2+^ levels and mitochondrial dysfunction suggesting a role of MAMs in the P2Et induction of apoptosis. Together, these observations suggest that P2Et could be advantageous in respect to other ICD inducers, because of Ecto-CRT and HMGB1 release would be more associated with Ca^2+^ alterations than ROS production. However, additional experiments using P2Et as ICD inducer should be done to evaluate the role of MAMs and Ca^2+^ in Ecto-CRT and ATP and HMGB1 release. However, additional studies to evaluate the role of PERK in P2Et antitumor activities should be validated using animal models.

In conclusion, our results suggest that plant extracts rich in polyphenols like P2ET induce ER-stress signaling through PERK phosphorylation which can modulate Ca^2+^ levels, induce apoptosis and immunogenic signals, showing future directions for the development of more efficient therapies that increase tumor immune response.

## Materials and methods

### Plant material

*C. spinosa* pods were collected in Villa de Leyva, Boyacá, Colombia. The plant was identified by Luis Carlos Jimenez, from the Colombian National Herbarium (voucher specimen number COL 523714. Colombian Environmental Ministry agreement number 220/2018 related to the use of genetic resources and derived products). The P2Et extract was produced under GMP conditions and chemically characterized as previously described^35,51^. In each assay, lyophilized P2Et was diluted in 95% ethanol obtaining a 25 mg/ml fresh solution.

### Reagents and antibodies

Propidium Iodure solution (Sigma-Aldrich St. Louis, MO, USA), FITC-Annexin V, ER-tracker^™^ green, MitoSOX^™^ red, MitoTracker^®^ red DiOC2(3) (3,3′-Diethyloxacarbocyanine Iodide), Fluo-4/AM, Fluo-3/AM, and Mag-Fluo-4/AM from Invitrogen/Molecular Probes (Chelmsford, MA, USA). Calcium chelator 1,2-*bis*-(2-aminophenoxy) ethane-N,N,N′,N-tetra acetic acid tetra (acetoxymethyl) ester (BAPTA/AM), PERK inhibitor I (GSK2606414), salubrinal, tauroursodeoxycholic acid (TUDCA), N-acetyl-l-cysteine (NAC), (±)-6-Hydroxy-2,5,7,8-tetramethylchromane-2-carboxylic acid (TROLOX), l-sulforaphane, MitoTEMPO, and quinacrine were purchased from Sigma-Aldrich (St. Louis, MO, USA). Doxorubicin hydrochloride (MP biomedicals, Solon, OH, USA). Antibodies against total and phosphorylated PERK (PERK 3192, p-PERK 3179), total IRE1α (3294), ATF-4 (11815), caspase 3 and 7 (9662 and 9494), XBP-1s (12782), and caspase 3 and 7 (9662, 9494) were obtained from Cell Signaling Technology (Beverly, MA, USA). Calreticulin (2907), phosphorylated IRE1α (p-IRE1, 48187), and phosphorylated eIF2α (p-eIF2α, 32157) antibodies were obtained from Abcam (Cambridge, MA, USA). ATF-6 antibody (40256SS) from Novus biologicals (Centennial, CO, USA). β-actin (AC-74), vinculin (V284), and GAPDH (G8795) antibodies were purchased from Sigma-Aldrich (St. Louis, MO, USA). Chop (sc-193) and HMGB1 (sc-74085) antibodies were obtained from Santa Cruz Biotechnology (Santa Cruz, CA, USA). EIF2α (eIF2α) antibody was obtained from Life Technologies (Chelmsford, MA, USA). CRISPR/Cas9 All-in-one Lentivectors were purchased from Applied Biological Materials (Richmond, BC, Canada). Lipofectamine 2000^®^ was used for all transfections (Invitrogen) and Puromycin for selection (Gibco, life technologies, NY, USA).

### Cell line and culture conditions

For all experiments, melanoma B16-F10 cell line (American Type Culture Collection, Manassas, VA, USA) was cultured in RPMI-1640 (Gibco, Life Technologies, NY, USA). The medium was supplemented with heat-inactivated fetal calf serum (10%), 2 mmol/L l-glutamine, 100 U/mL penicillin, and 100 μg/mL streptomycin, and 25 mmol/L Hepes buffer (Gibco, Life Technologies, NY, USA). Cells were incubated in a humidified environment at 37 °C and 5% CO_2_ and grown until 75% confluence. Cells were collected using trypsin/EDTA (ethylenediamine-tetra-acetic acid) 0.25% phenol red (Gibco, Life Technologies, NY, USA). For treatments, 3 × 10^5^ cell were seeded on six-well plates, incubated for 12 h and treated as indicated in each assay. The treatment cells were done for different periods using IC50 P2Et extract (74.7 µg/ml).

### CRISPR/Cas9-mediated genomic editing

For CRISPR clones, knockout was generated using sgRNAs in CRISPR/Cas9 All-in-one Lentivectors purchased from Applied Biological Materials (ABM) (Richmond, BC, Canada). Mouse Eif2ak3 (PERK) (three targets, K4522205), Xbp1 (Target 2, K4387407) and Scrambled sgRNA CRISPR/Cas9 All-in-One Lentivector (K010) were used for transfection of 3 × 10^5^ B16-F10 cells. Cells were plated in six-well plates and transfected using Lipofectamine^®^ 2000 with target or scramble plasmids following the manufacturer’s protocol. The medium was exchanged with complete medium after overnight incubation and selected one week with puromycin 2.5 µg/ml. Resistant cells were plated in 96-well plates by limiting dilution to generated single cell clones and expanded. Protein expression was evaluated by western blot for each single cell clone.

### Western Blot

Cells lysates protein concentration was measured by the Micro BCA^™^ protein assay (Thermo Scientific, Chelmsford, MA, USA) and electrophoresed in 10% Tris-Glycine gels, transferred to polyvinylidene fluoride membranes using standard techniques, and immunoblotted with the corresponding primary and secondary antibodies. Membrane-bound immune complexes were visualized using ECL (Thermo Scientific, Chelmsford, MA, USA) in a Chemi-Doc imaging system (Bio-Rad). Densitometry of each protein normalized to vinculin or β-actin was calculated using the Bio-Rad Image-Lab software.

### Flow cytometry

#### Cell death evaluation

PS externalization was assessed using Annexin V-FITC/PI. B16-F10 wild type or modified cells (3 × 10^5^) were treated with vehicle (ethanol), doxorubicin (a positive control), or P2Et for 6, 12, 24, or 48 h. In some cases, before treatment cells were incubated with different inhibitors for 2 h, as explained for each assay. After treatment, cells were suspended in Annexin buffer 1×, Becton Dickinson Biosciences (BD Biosciences, San Jose, CA) and incubated with Annexin V-FITC/PI for 15 min at RT.

#### Ecto-calreticulin evaluation

B16-F10 wild-type or modified cells (3 × 10^5^) were plated in 6-well plates and treated with vehicle (ethanol), doxorubicin (a positive control) or P2Et for 12 h. Cells were stained as we previously described^2^.

#### Vesicular ATP evaluation

B16-F10 wild type or modified cells (3 × 10^5^) were plated in 6-well plates and treated with vehicle (ethanol), mitoxantrone (a positive control) or P2Et for 48 h. Cells were collected and stained as was previously described^31^. Briefly, quinacrine was prepared to 1 μM final concentration in Krebs–Ringer solution (125 mM NaCl, 5 mM KCl, 1 mM MgSO_4_, 2 mM CaCl_2_, 6 mM glucose, and 25 mM HEPES buffer, pH adjusts 7.4). Cells were loaded with quinacrine warm solution for 30 min at 37 °C, washed and resuspended in 1 mg/ml of PI solution.

Samples were acquired with FACS Aria II (BD Immunocytometry Systems, San Josè, CA, USA) and Cytoflex (Beckman Couler, Waltham, MA, USA) flow cytometers. All data experiments were analyzed by FlowJo software (Ashland, OR, USA).

### ER stress evaluation

During ER stress, an expansion of ER must adjust to the needs of the cell and it is necessary to increase chaperones production. ER-membrane expansion was evaluated as a measure of ER stress. Thus, B16-F10 cells treated were collected and labeled with 0.1 µM ER-Tracker green dissolved in HBSS for 30 min at 37 °C and analyzed by FACS. Following western blotting protocol describe before, membranes were immunoblotted with PERK, p-PERK, IRE 1, p-IRE 1, eIF2α, p-eIF2α, ATF-4, CHOP, or ATF-6 antibodies.

### Measurement of mitochondrial membrane potential

To evaluate mitochondrial integrity B16-F10 cells were labeled for 15 min with MitoTracker (0.5 µM) or MitoSOX (5 µM) at 37 °C and analyzed by FACS. Loss of mitochondrial membrane potential was measured by FACS using DiOC_2_(3) and Jc-1 probes. Treated cells were stained with DiOC_2_(3) at final concentration of 50 nM in PBS for 30 min at 37 °C, as a control for mitochondria uncoupling B16-F10 cells were treated 5 min prior FACS analysis with carbonilcianuro-m-clorofenilhidrazona (CCCP) which decrease the DiOC_2_(3) retention (DiOC^low^ cells). Jc-1 2.5 µg/ml solution was prepared in warm RPMI-1640 without phenol red and added to 1 ml of cells at 37 °C for 10 min. Totally, 20,000 cells were analyzed by FACS. Jc-1 aggregates were evaluated in FL-2 (585 nm), showing a normal mitochondrial membrane potential, while an increase in FL-1 (530 nm) fluorescence is associated with monomers due to loss of the mitochondrial membrane potential.

### Seahorse analysis

SCR o PERK B16 KO cells were collected after treatment with P2Et IC50 or Vehicle for 12 h, seeded in Seahorse XF96 plate coated with CellTak (Corning) and incubate in normal condition for 4 h. After cells were washed with XF Assay Media from SeaHorse Bioscience containing 25 mM glucose, 2mM l-glutamine, and 1 mM sodium pyruvate and incubated at 37 °C without CO_2_ for 20 min. The XF Cell Mito Stress test kit (Agilent) was performed according to the manufacturer's instructions with measurement basal or in response to oligomycin (1 μM), carbonyl cyanide-p-(trifluromethoxy) phenylhydrazone (FCCP, 1 μM), and rotenone/antimycin injection (0.5 μM each).

### Calcium assays

B16-F10 cells treated were collected and stained as previously described by Bidaux et al.^52^. Briefly, cells were washed with physiological salt solution (PSS, 140 mM NaCl, 5 mM KCl, 1 mM MgCl_2_, 10 mM glucose, 10 mM Hepes, and pH adjusted to 7.4 with NaOH) without calcium. Cells were labeled with membrane-permeable calcium AM esters Fluo-3 (1.5 μM) or Fluo-4 (1.5 μM) diluted in PSS supplemented with 70 μM CaCl_2_ and 0.05% (v/v) Pluronic F-127 (Sigma-Aldrich, St. Louis, MO, USA) and incubated 30 min at 37 °C. After that, cells were washed twice and incubated at 37 °C in PSS (70 μM CaCl_2_) for 30 min. Cells were centrifuged and stabilized incubating in PSS supplemented with a high concentration of CaCl_2_ (1.7 mM) at RT for 30 min and immediately analyzed by FACS.

### Confocal microscopy

For immunofluorescence assays, cells were cultured in collagen treated-Fisher-Brand Microscope Cover Glass in 12-well plates. For HMGB1, 1.5 × 10^4^ cells were seeded onto coverslips and were grown overnight before treatment. Cells were treated for 48 h with P2Et, Doxorubicin or vehicles and washed with PBS twice. Cells were fixed in 4% formaldehyde for 20 min, washed and permeabilized with 0.1% Triton X-100 for 5 min. This was followed by blocking with 10% FCS PBS for 1 h, and incubation with anti-HMGB1 primary antibody for 30 min at room temperature (RT) and Alexa Fluor 488- conjugated secondary antibody for 30 min. Finally, cells were stained with DAPI (300 nM) for 5 min and coverslips were mounted on slides with Prolong Antifade Reagent (Life Technologies, Woburn, MA, USA). Images of 640 × 640 pixels resolution were acquired with a laser scanning confocal microscope FV1000 (Olympus, Tokyo, Japan) using UPLSAPO 60× 1.35 NA oil immersion objective.

### Statistical analysis

Statistical analysis of the significance between two groups was calculating using two-tailed unpaired and paired Student’s *t* test for most of the statistical analyses and a *p* value of <0.05 was considered statistically significant. The specific statistical test results are indicated in each figure: ^*^p < 0.05; ^**^p < 0.01; ^***^p < 0.001; ^****^p < 0.001. Statistical analyses were performed in GraphPad Prism version 8.0.

## Supplementary information

Figure Supp 1

Figure Supp 2

Figure Supp 3

Supplemental Material File #1
