# Supplementary figures and images for: Polyphenol-rich extract induces apoptosis with immunogenic markers in melanoma cells through the ER stress-associated kinase PERK
Source: Cell Death Discov. 2019 Sep 9;5:134. doi: 10.1038/s41420-019-0214-2 (PMC6733947; doi:10.1038/s41420-019-0214-2)

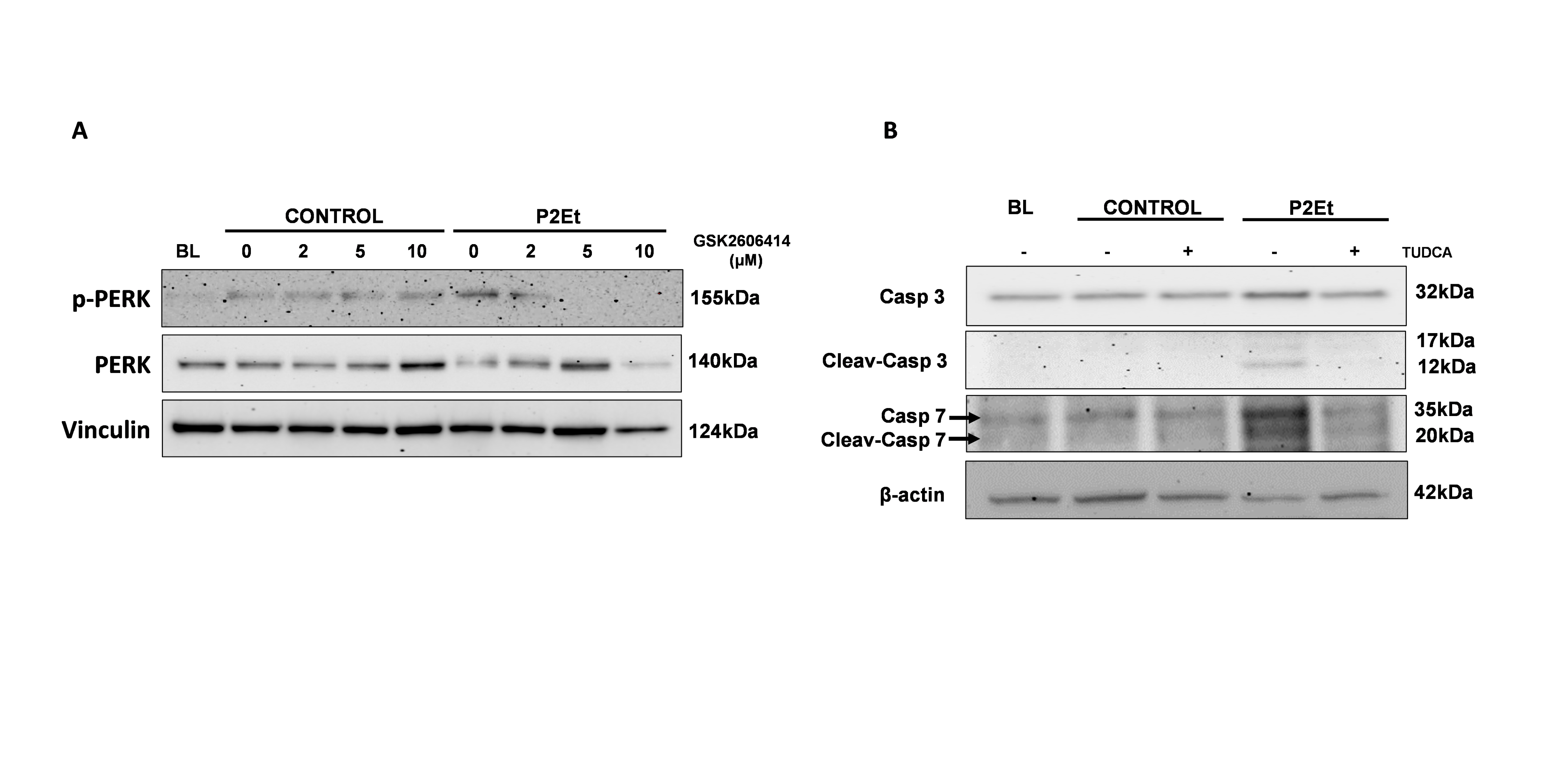

Supplement: Supplementary file 1 — Figure Supp 1 [file 41420_2019_214_MOESM1_ESM.tif]

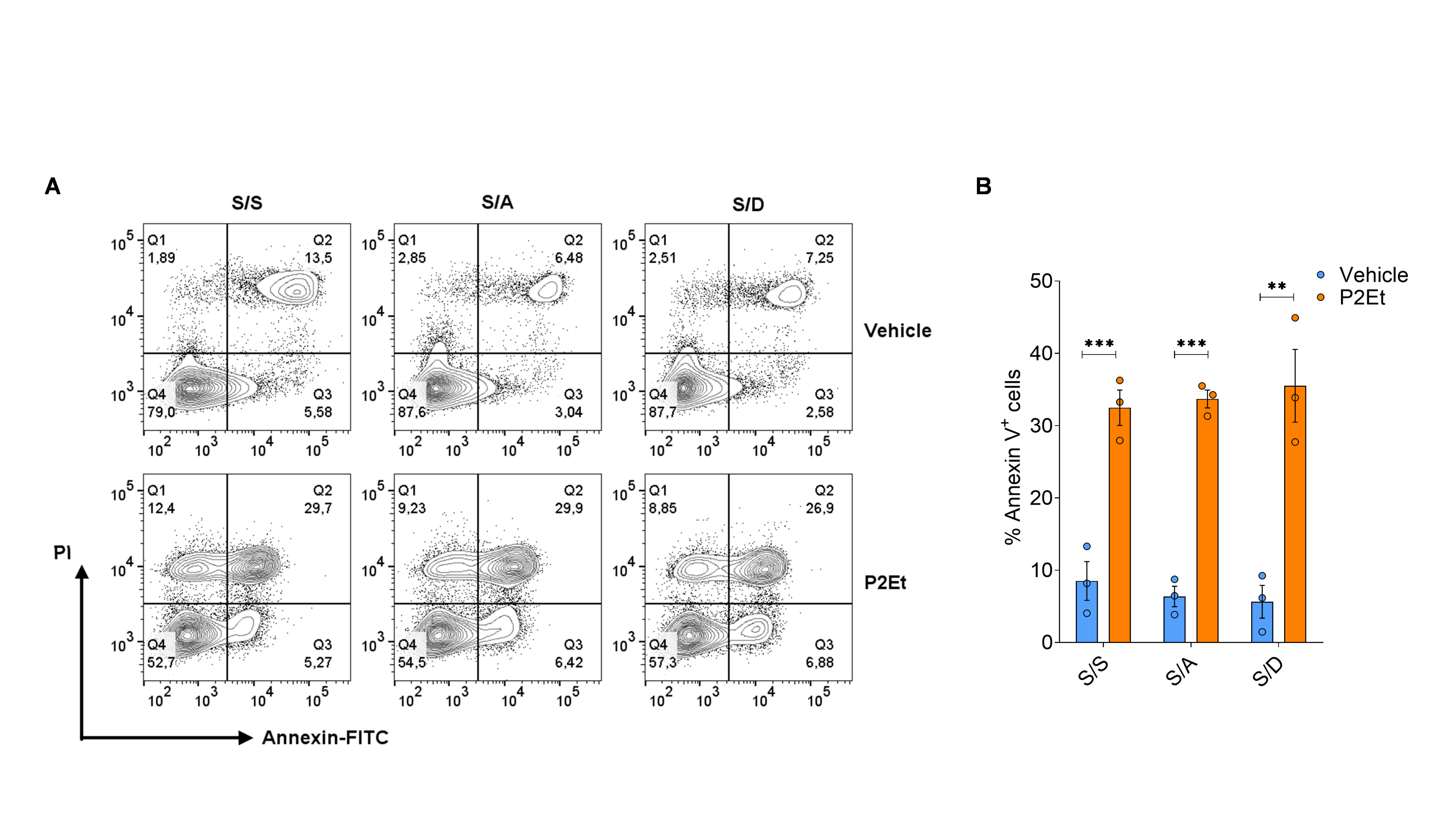

Supplement: Supplementary file 2 — Figure Supp 2 [file 41420_2019_214_MOESM2_ESM.tif]

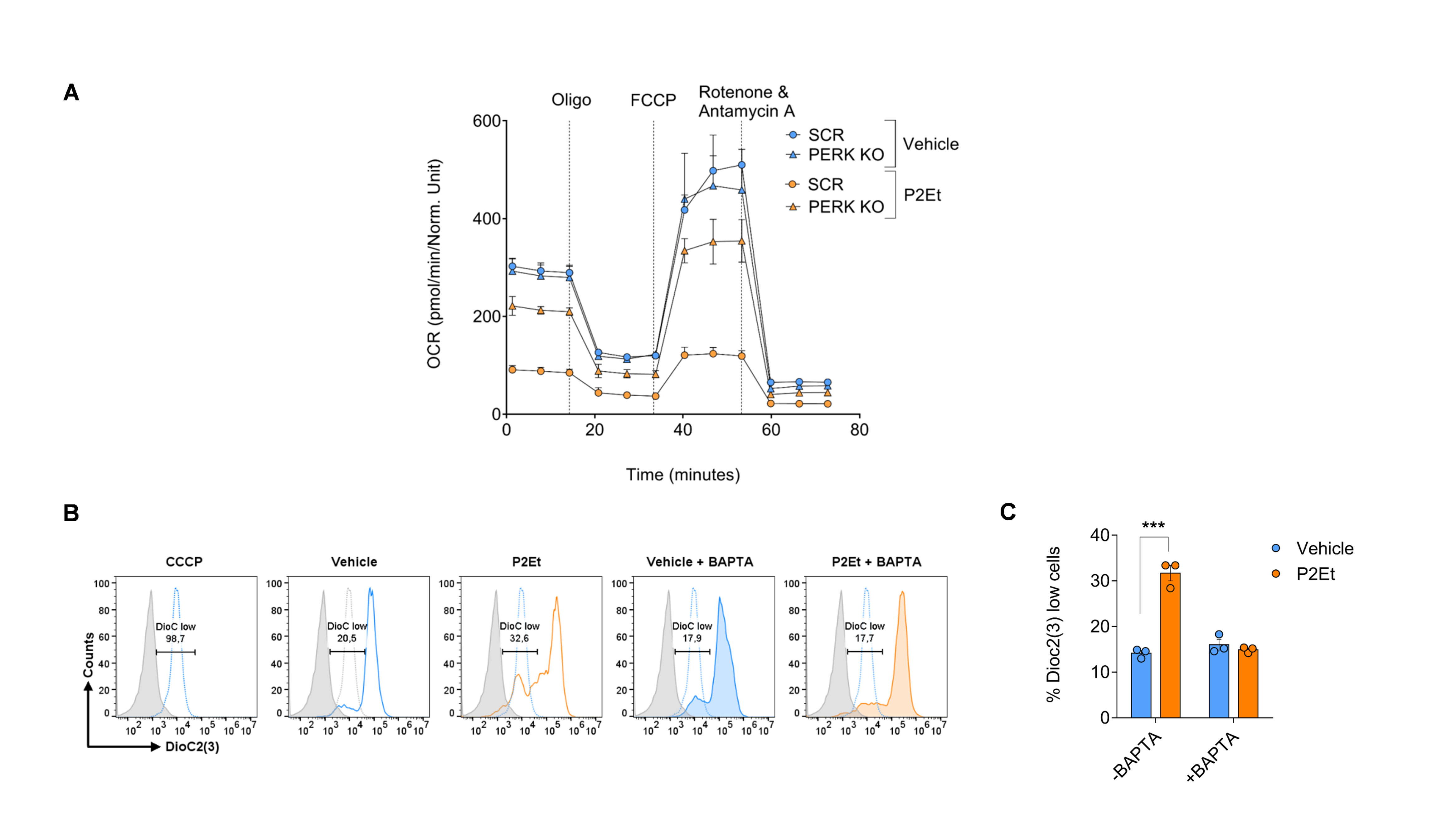

Supplement: Supplementary file 3 — Figure Supp 3 [file 41420_2019_214_MOESM3_ESM.tif]
