# Supplementary material for: Polyphenol-rich extract induces apoptosis with immunogenic markers in melanoma cells through the ER stress-associated kinase PERK
Source: Cell Death Discov. 2019 Sep 9;5:134. doi: 10.1038/s41420-019-0214-2 (PMC6733947; doi:10.1038/s41420-019-0214-2)
Supplement: Supplementary file 4 — Supplemental Material File #1 [file 41420_2019_214_MOESM4_ESM.docx]

**Supplementary Fig. 1. Endoplasmic reticulum inhibitors decrease caspase activation and PERK-phosphorylation in B16-F10 cells treated with P2Et.** B16-F10 cells were pretreated 2 hours with ER stress inhibitors and then treated with P2Et IC50 or Vehicle for additional 24h. **A.** Cleavage evaluation of caspase-3 and 7 by western blot analysis in B16-F10 cells pre-treated with 0.5mM TUDCA. **B.** B16-F10 cells pretreated with several concentrations of GSK2606414 (2µM, 5µM, and 10µM) were analyzed by western blot.

**Supplementary Fig. 2. P2Et extract induces apoptosis in an eIF2α-phosphorylation independent manner. A.** B16-F10 stably transfected cells with p-eIF2α plasmids coding to serine 51 mutant (S/S, S/A or S/D) were treated with P2Et or vehicle for 24h. Representative contour plot of Annexin V/PI stain is shown. **B.** Percentages of Annexin V positive cells were expressed as mean±SEM of three independent experiments.

**Supplementary Fig. 3. P2Et extract decreases mitochondrial respiratory function through calcium modulation. A.** Oxygen consumption rate (OCR) profile of SCR and PERK KO cells treated with P2Et IC50 or Vehicle for 12h. **B.** B16-F10 cells were pre-treated with 10µM BAPTA for 2h and then treated with P2Et IC50 or vehicle for additional 24h. Cells were labeled with DioC2(3), the gate to analyze was made over B16-F10 cells treated with 1µM of carbonilcianuro-m-clorofenilhidrazona (CCCP) 5min prior FACS. A representative histogram of DioC2(3) fluorescence is shown **C.** Percentage of DioC2(3) low cells expressed as mean±SEM of three independent experiments is shown
